# Antioxidant, Anti-Inflammatory, and Antidiabetic Activities of Leaves and Stems of *Uapaca bojeri* Bail. (*EUPHORBIACEAE*), an Endemic Plant of Madagascar

**DOI:** 10.3390/ph13040071

**Published:** 2020-04-17

**Authors:** Zoarilala Rinah Razafindrakoto, Dario Donno, Nantenaina Tombozara, Harilala Andriamaniraka, Charles Andrianjara, David Ramanitrahasimbola, Gabriele Loris Beccaro

**Affiliations:** 1 Institut Malgache de Recherches Appliquées, B.P. 3833, 101 Antananarivo, Madagascar; zo_ari_lala@yahoo.fr (Z.R.R.); nzara89@gmail.com (N.T.); charles.andrianjara@gmail.com (C.A.); dramanitrahasimbola@yahoo.fr (D.R.); 2 Dipartimento di Scienze Agrarie, Forestali e Alimentari, Università degli Studi di Torino, 10095 Grugliasco (TO), Italy; gabriele.beccaro@unito.it; 3 Organic Chemistry Department, Faculty of Sciences, University of Antananarivo, P.O. Box 566, Antananarivo 101, Madagascar; 4 Ecole Supérieure des Sciences Agronomiques, Université d’Antananarivo, P.O. Box 566, Antananarivo 101, Madagascar; jharilala@gmail.com; 5 Pharmacy Department, Faculty of Medicine, University of Antananarivo, P.O. Box 566, Antananarivo 101, Madagascar

**Keywords:** tapia, endemism, pharmacological studies, HPLC, phytochemical fingerprint

## Abstract

*Uapaca bojeri* is an endemic Malagasy plant used by the local population. This work aimed to evaluate antioxidant, anti-inflammatory, and antidiabetic activities of the methanol extracts of *U. bojeri* leaves and stems and to report their total phenolic content and the bioactive compound content by HPLC methods. Antioxidant capacity was determined by DPPH and ferric reducing antioxidant power (FRAP) assays. An in vivo carrageenan-induced paw oedema and acetic acid-induced writhing test in mice were used for anti-inflammatory activity evaluation. An oral glucose tolerance test was performed in mice to evaluate antidiabetic activity. The total bioactive compound content of leaves was higher than that of stems. Stem methanol extract inhibited the free radical DPPH more than the leaf methanol extract. Leaf methanol extract inhibited, in a dose-dependent manner, the carrageenan-induced paw oedema more than the stem extract, but their inhibition of the pain symptoms caused an acetic acid-induced decrease similar to the number of writhes in the dose-dependent case. The leaf and stem methanol extracts significantly reduced blood glucose levels after 30 min of glucose loading in mice compared to the control group blood glucose reduction. The presence of several bioactive compounds in *U. bojeri* contributed to the different biological activities, but isolation and identification of these bioactive molecules are necessary to confirm these pharmacological properties.

## 1. Introduction

Madagascar is well-known for its high flora endemism rate that has been estimated at about 80% of total plant species (from 12,000 to 14,000) [1], but only 10% of these have been biologically investigated [2]. On the other hand, traditional medicine plays an important role in the Malagasy health system and endemic Malagasy plants are supposed to be a source of new chemical compounds with a specific interesting pharmacological profile and therapeutic uses. Several Malagasy plants and their traditional uses are registered in Malagasy Pharmacopeia. Among these plants, *Uapaca bojeri* Bail., known as “tapia” by the local population, is still utilized for its potential activity against diabetes mellitus, infectious diseases, and hypertension [3]. Leaf infusion of the species is often used by the local population to treat headache and dizziness, to decrease hypertension, and as a cardiac tonic. Stem decoctions are used to alleviate stomach affection and to treat diarrhea. The infusion is also consumed for diabetes treatment. Ripe fruits are eaten as complementary food.

*U. bojeri* is a tree belonging to the *Euphorbiaceae* family. This plant is widespread in the four Tapia forests located in three regions of Madagascar, including the hill of Imamo in the Itasy region, the hills of Manandona and Itremo in the Imoromania region, and the hill of Isalo in the Horombe region [4]. This endemic plant is locally used as a host for the Malagasy silkworms called landibe (*Borocera cajani*) for textile production [5].

Tapia forests are the most xerophytic evergreen forests of the Madagascar highlands. The mean annual temperature in these ecosystems ranges between 17 °C and 22 °C. The rainfall cycle is characterized by an average annual precipitation of about 1482 mm. The soil mainly comprises silica and alumina with a pH value of 5. The soil textures are ferralitic, clay-sandy, and strongly desaturated [6]. The Tapia forests are characterized by upper strata trees with low branching, as *U. bojeri*, and lower strata of suffrutescent plants and shrubs [4]. The genus *Uapaca* is represented by 60 species in Africa and 12 species are endemic in Madagascar. *U. bojeri* is a tree ranging in height from 3 to 5 m, but it can sometimes reach from 10 to 12 m, with cracked woody bark and dense foliage. Leaves are simple, sclerophyllous, alternately tightly spiraled, and obovate in shape of the spatula (9 cm × 4 cm), with a dark green upper surface. Petioles are very short and covered with glands. The flowering season is between March and September. Its fruit is a drupe that appears in October [7].

This work aimed to evaluate the antioxidant capacity and the antalgic, anti-inflammatory, and antidiabetic activities of the methanol extracts of leaves and stems of *U. bojeri*. This study also reported the total phenolic content (TPC) and the total bioactive compound content (TBCC) and their synergistic contribution to these properties. According to our knowledge, this study is the first report on the phytochemistry and pharmacology of this Malagasy endemic plant in order to confirm some traditional uses of this plant.

## 2. Results and Discussion

### 2.1. Total Bioactive Compound Content (TBCC) and TPC

In this study, 34 bioactive compound and nutritional substances (Table 1) have been selected as markers for the determination of HPLC fingerprints because of their importance in humans [8,9]. The amounts of each bioactive compound for each plant part are reported in Table 1. The sum of the amount of each detected compound represents the TBCC detected in each plant extract.

The number of compounds identified in leaves is higher than those identified in stems (9 vs. 6). Moreover, the amount of each detected bioactive compound is higher in leaves than in stems except for the caffeic acid (0.77 ± 0.45 vs. 2.08 ± 0.07 mg/100 g of dry weight (DW)); this compound is the only molecule quantified in the cinnamic acid class. Hyperoside (77.94 ± 6.37 mg/100 g of DW), quercetin (151.92 ± 13.35 mg/100 g of DW), and rutin (72.20 ± 7.97 mg/100g of DW) were quantified in leaves, while no flavonol was detected in stems. Flavonols in the stems may not show the same structures of selected markers. This study showed few differences between ellagic acid quantified in leaves and stems, but the leaves (338.04 ± 123.79 mg/100 g of DW) presented a higher value than the stems (335.14 ± 106.96 mg/100 g of DW). Castalagin was detected both in the leaves (943.83 ± 98.80 mg/100 g of DW) and stems (406.35 ± 223.20 mg/100 g of DW). Compared to the results of Rakotoniaina et al. [10] on *Chrysophyllum boivinianum*, the number of identified compounds is the moiety either in the leaf (9 vs. 18) or in the stem (6 vs. 12). Only hyperoside was detected in leaf of the *C. boivinianum* (8.89 ± 2.60 mg/100g of DW), which confirms that the leaf and stem of *U. bojeri* could be good sources of flavonols. On the one hand, the amount of ellagic acid seems to be the same for leaf extracts of both plants (338.04 ± 123.79 vs. 385.15 ± 112.28 mg/100g of DW). On the other hand, those in stem extracts are totally different (335.14 ± 106.96 vs. 167.32 ± 34.60 mg/100 g of DW). The TPC by spectrophotometric analysis of *U. bojeri* is reported in Table 4. The stem extract value was higher than the leaf one (5854.17 ± 1247.65 vs. 3624.72 ± 268.07 mg gallic acid equivalents (GAE)/100 g of DW). These results were statistically significant (*p* < 0.05). These values are significantly high when compared to the other methanol extracts of Malagasy endemic plants such as *Catharanthus roseus* with a value of 959.00 ± 11.50 mg GAE/100 g of fresh weight [11], and *C. boivinianum* with values of 805.16 ± 1.08 and 249.12 ± 7.11 mg/100 g of DW respectively for the leaf and stem [10]. Polyphenolic compounds are phytochemicals with high antioxidant capacity. The relationships between antioxidant capacity and phenolic content have been reported earlier by Newell et al. [12]. Flavonols and phenolic acids (as caffeic acid) can inhibit in vitro low-density lipoprotein oxidation quenching active oxygen species and reducing thrombotic tendency [9,10]. Flavonoids may attenuate inflammation, modulate arachidonic acid metabolism, and inhibit cyclooxygenase. Moreover, many natural phenolic compounds have also shown anti-carcinogenic activity in several animal models [10].

Succinic acid was the only organic acid detected in all the samples. The succinic acid level was 533.74 ± 340.08 mg/100 g of DW for stems and 1275.65 ± 434.99 mg/100 g of DW for leaves. Statistical analysis showed a significant difference (*p* < 0.05) between the succinic acid amounts in the two considered samples. The anti-inflammatory activity of succinic acid derivatives was reported by Chien et al. [13]; these compounds are involved in the potential immunomodulatory action against RAW264.7 macrophage cells. These compounds significantly increased spontaneous tumor necrosis factor-alfa (TNF-α) secretion from unstimulated RAW264.7 cells, but they suppressed IL-6 production in LPS-stimulated cells, suggesting that they may show an anti-inflammatory effect on macrophage-mediated responses [13].

Carotenoids were mostly represented by lycopene, which showed the second highest level (1015.44 ± 253.01 mg/100 g of DW) among all the selected markers, lutein (20.99 ± 1.34 mg/100 g of DW), *β*-cryptoxanthine (12.55 ± 6.54 mg/100 g of DW), and traces of zeaxanthine (0.46 ± 0.03 mg/100 g of DW) in leaves, while the stems contained only some traces of lycopene (1.79 ± 0.95 mg/100 g of DW), lutein (2.85 ± 0.07 mg/100 g of DW), and *β*-cryptoxanthine (0.78 ± 0.34 mg/100 g of DW). HPLC analysis of different parts of *U. bojeri* showed that the plant may be a good source of bioactive compounds, even if no monoterpenes (except traces of sabinene in leaves and vitamin C) were identified. According to Hernandez-Ortega et al. [14], guajillo, pasilla, and ancho peppers showed good antioxidant capacity and a significant peripheral analgesic activity at different doses, and they may be useful for inflammation and pain relief as an anti-inflammatory due to the high level of carotenoids in these plants. The results suggested that carotenoids, particularly lycopene and lutein, show significant anti-inflammatory and analgesic benefits, and they could be useful for pain and inflammation relief.

Polyphenols represent the most important class of bioactive compounds involved in biological activities of the considered plant material. In any case, the optimization of *U. bojeri* pharmacological activities may be due to the additive and synergistic effects of several secondary metabolites (called phytocomplex) [15]. Phytocomplex was intended as the sum of specific markers with demonstrated health-promoting activities that can be identified by the comparison between spectroscopic data/retention times and authentic standards (external standard calibration method), following the “multi-marker approach.” The quantification of few active components (“marker approach”) is one of the most important techniques used for the plant material characterization. As the biological properties are due to more than one or two single chemical compounds, the “multi-marker approach” is the extension of the “marker approach.” In this study, all the identified molecules (phytochemical profile) are used to represent the whole sample, as already performed for many complex systems (e.g., herbal preparations, herbs, foodstuffs, food supplements) [9]. It is not simple to consider all the bioactive molecules that may be part of the phytocomplex because of their high number, but, thanks to the multi-marker approach, the phytocomplex may be approximated to the most important substances with biological properties.). For this reason, five phenolic classes were selected for the evaluation. Moreover, levels of carotenoids, vitamin C, organic acids, and monoterpenes were also studied.

### 2.2. In Vitro Antioxidant Capacity

This preliminary study aimed to evaluate the phytocomplex biological properties rather than the pharmacological activities of specific molecules or groups of bioactive compounds. The pharmacological activity and antioxidant capacity of a phytocomplex are due to synergistic and additive effects of all the molecules (phytochemicals, nutritional substances, peptides, etc.) present in a plant material.

The antioxidant activity of the *U. bojeri* stems and leaves was determined by measuring the free radical scavenging activity (DPPH) and the ferric reducing antioxidant power (FRAP). All the antioxidant activity results are reported in Table 2. Although the FRAP and DPPH protocol are an in vitro chemical-based assay without large application for cell models (antioxidant capacity involves modulation of redox cell signaling, up-regulation of detoxifying and antioxidant enzymes, and gene expression besides scavenging free radicals), these tests may evaluate the potential of a plant material as an inhibitor of a target substance oxidation. Moreover, these methods are utilized for screening low cost analysis and they yield an index value to order and compare several plant samples or natural products. The DPPH assay is based on the ability of specific compounds (H-A) in the extracts to leave hydrogen to be fixed by the free radical DPPH^•^ (purple color) and to form a new stable radical and the hydrogenated form DPPH-H (yellow color) according to the following reaction: DPPH^•^ + H-A → DPPH-H + A^•^. This color change is accompanied by an absorbance decrease of the H-A amount [16]. The IC_50_ of the ethanol extracts and gallic acid was reported in Table 2. Methanol extracts’ antioxidant activity was confirmed by FRAP results with values of 70.17 ± 9.53 and 69.20 ± 1.41 mmol Fe^2+^/kg of DW respectively for the stem and leaf. These values could be considered as higher values compared to the other studies: 27.60 ± 0.32 and 19.86 ± 7.35 mmol Fe^2+^/kg of DW for the leaf and stem extracts of *C. boivinianum*, respectively [10]. The stem extracts reduced DPPH more than the leaf extracts with the IC_50_ values of 33.32 ± 0.69 vs. 47.36 ± 3.00 µg/mL (*p* < 0.0001), but their capacity to reduce ferric ions are similar (*p* < 0.05). It should be due to the presence of high TPC amounts in all the extracts. Several studies reported the relationship between antioxidant activity and phenolic compounds [17,18,19].

### 2.3. Anti-Inflammatory Activity

#### 2.3.1. Carrageenan-Induced Paw Oedema

The carrageenan-induced hind paw oedema model is a widely used anti-inflammatory screening protocol for natural products [20]. This protocol is biphasic. The first phase involves histamine and serotonin release for 2 h, while the second phase is mediated by prostaglandins. The oedema reduction rate produced by leaf and stem methanol extracts of *U. bojeri* is reported in Table 3. The leaf and stem methanol extracts of *U. bojeri* showed a significant (*p* < 0.001) effect, at a dose of 200 and 400 mg/kg, on the reduction of carrageenan-induced inflammation in mice after 2 h compared to the control group (I). Moreover, this oedema reduction is in a dose-dependent manner (*p* < 0.001) with ANOVA. The methanol extracts also exhibited a significant dose-dependent inhibition (*p* < 0.001) on the delayed steps of the oedematogenic response (3 h after the edema induction). Indomethacin very significantly (*p* < 0.01) inhibited the carrageenan-induced oedema after 3 h with a reduction of about 86.83%. Indomethacin is a known cyclooxygenase inhibitor able to attenuate the carrageenan response in the inflammation second phase, mainly prostaglandin-mediated [21,22].

Thus, as shown in Table 3, the mean percent of anti-inflammatory effects (83.62% and 71.59% at a dose of 400 mg/kg), showed by the leaf and stem extract of *U. bojeri*, was particularly high on the delayed steps (3 h) of the carrageenan-induced oedematogenic response when compared to the control sample. As the anti-inflammatory effect of the methanol extracts of *U. bojeri*, as indomethacin, was mainly observed in the second phase of inflammation, it suggests that the anti-inflammatory activity could be due to the prostaglandin release inhibition. However, the early anti-inflammatory effect of the leaf methanol extract of *U. bojeri* (43.80% and 44.71%, respectively at the doses of 200 and 400 mg/kg), at the first period (1 h), against carrageenan-induced inflammation due to histamine, serotonin, and quinine-like substances release, should not be ruled out.

#### 2.3.2. Acetic Acid-Induced Writhing

The analgesic activity of the *U. bojeri* extracts was evaluated by the writhing method, the most common peripheral analgesic animal model for the screening of analgesic drugs [23]. The number of writhes and the percentage of inhibition produced by leaf and stem methanol extracts of *U. bojeri* are reported in Table 4. The intraperitoneal injection of 1% acetic acid solution into the control group caused 24.90 ± 2.25 writhing between the intervals of 25 min after the 5th min of acetic acid induction. Mice treated with the *U. bojeri* leaf and stem extracts showed, in the dose-dependent manner (*p* < 0.001 with ANOVA), a significant reduction in the number of writhes (*p* < 0.05) compared to the control group (Table 5). The indomethacin inhibited the acetic acid induction at 84.34% for a number of writhing reductions to 3.90 ± 1.12 (*p* < 0.001). The inhibition of lipoxygenases and/or cyclo-oxygenases (and other inflammatory mediators) may mediate the peripheral analgesic effect, while the inhibition of central pain receptors could mediate the central analgesic action.

### 2.4. Antidiabetic Activity

An oral glucose tolerance test was used for the determination of the preliminary antidiabetic activity of *U. bojeri* methanol extracts. As shown in Table 5, the negative control group presented the highest blood glucose level at any time after the glucose loading. The glycaemia increased after the first 15 min, and it then decreased thereafter for each group, except for the group receiving stem extracts that reached the maximum level of glycaemia at 30 min, indicating that the body released the insulin hormone to break glucose as a homeostatic process. The diminution of the blood glucose level increased in a dose-dependent manner for both methanol extracts of *U. bojeri*. The leaf and stem extracts, at a dose of 200 mg/kg, exert a similar activity as glibenclamide, but at a dose of 400 mg/kg, they reduce the blood glucose level more than the positive control 30 min after the glucose loading. This means that leaf and stem extracts showed a high hypoglycemic activity. At 120 min, the leaf and stem extracts reduce the blood glucose level in a dose-dependent way. Compared to the negative control group, leaf and stem extracts (200 and 400 mg/kg) and glybenclamide (10 mg/kg) produced a significant decrease (*p* < 0.05) in the plasma glycaemia from 30 to 120 min after glucose administration. Benzoic acid-related molecules enhanced insulin effects and inhibited insulinase, as shown in other studies [24]. Phenolic compounds, as molecules identified in this research, are natural compounds with antioxidant activity and are used as anti-diabetic drugs. Flavonoids are natural phenolics with several health-promoting properties. Phenolic compounds are generally known for their strong antioxidant properties [25]. Some antioxidant molecules have been reported to be potent α-glucosidase inhibitors and also regulators of hyperglycemia (together to other diabetic complications) deriving from oxidative stress [26].

## 3. Materials and Methods

### 3.1. Plant Materials and Methanol Extract Preparation

Leaves and stems of *U. bojeri* were collected in the Tapia forest (Imamo, Arivonimamo District) in the Itasy region on July 2018. A specimen was identified by Mr. Benja Rakotonirina, the botanist of the *Institut Malgache de Recherches Appliquées* (IMRA), and a specimen voucher was compared to the previous reference TN-021/LPA in the IMRA Botany Department. The leaves and the stems were separately dried at room temperature in a cool and airy place, away of sunlight, before being ground. Then, 100 g of each powder sample was macerated with 300 mL in a mixture of methanol-water (95-05, v-v) for 24 h before filtration. Then, plant material was again extracted with the same solvent twice to obtain a better extraction efficiency. The filtrates were mixed and evaporated under reduced pressure at 40 °C using a rotary evaporator (Büchi R-114) coupled to a vacuum pump (PC 3001 VARIO^pro^) and a cooling system (UKT 600 LAUDA). The methanol extracts were stored at 4 °C until analysis.

### 3.2. Chemicals and Solvents

Sodium carbonate, Folin–Ciocalteu phenol reagent, sodium acetate, acetic acid, citric acid, potassium chloride, hydrochloric acid, iron(III) chloride hexahydrate, 2,4,6-tripyridyl-S-triazine, 1,2-phenylenediamine dihydrochloride (OPDA), all polyphenolic and terpenic standards, carotenoids (-carotene, -carotene, -cryptoxanthin, lutein, lycopene, zeaxanthin), potassium dihydrogen phosphate, phosphoric acid, glibenclamide, 1,1-diphenyl-2-picrylhydrazyl (DPPH), carrageenan, indomethacin, and HPLC-grade methanol and acetonitrile were purchased from Sigma-Aldrich (St. Louis, MO, USA). Acetic acid, ethanol, organic acids, and HPLC-grade formic acid were purchased from Fluka BioChemika, Buchs, Switzerland. Ethylenediaminetetraacetic acid disodium salt was purchased from AMRESCO (Solon, OH, USA). Sodium fluoride was purchased from Riedel-de Haen (Seelze, Germany). Cetyltrimethylammonium bromide (cetrimide), ascorbic acid (AA), and dehydroascorbic acid (DHAA) were purchased from Extrasynthèse (Genay, France). Milli-Q ultrapure water was produced by Sartorius Stedim Biotech mod. Arium (Sartorius, Göettingen, Germany).

### 3.3. Total Phenolic Content (TPC)

TPC was evaluated using the Folin–Ciocalteu reagent according to the method of Slinkard and Singleton [27]. In addition, 5 g of dried powder of leaves or stems was macerated for 24 h in 35 mL of methanol-water (95:5 v/v) solution acidified by hydrochloric acid in the dark. After filtration, 200 µL of the samples was added to 1 mL of the Folin–Ciocalteu reagent (dilution 1:10) and to 800 µL of 7.5% Na_2_CO_3_. The mixture was stored in the dark for 30 min before absorbance measurements at 765 nm.

Each experiment was carried out in triplicate and the results were expressed as mg gallic acid equivalents (GAE)/100 mg of dry weight (DW). Gallic acid standard solutions were prepared at 0.02–0.10 mg/mL.

### 3.4. Chromatographic Analysis

#### 3.4.1. Samples Preparation Protocol for HPLC Analysis

Extracts were prepared according to Donno et al. [28] in triplicate. Briefly, 5 g of plant powders were weighed using an electronic balance (PM460, Delta Range) and then macerated in 35 mL of methanol–water (95:5 v/v) solution acidified by hydrochloric acid for 24 h in the dark. Samples were then mixed and homogenized for 3 min using a mixer (Ultra Turrax T25 basic, IKA-Werke) before a second maceration (72 h). Extracts were again mixed and homogenized for 3 min, then placed in the dark. After 24 h, they were filtered using Whatman filter paper (Hardened Ashless Circles, 185 mm Ø) and the filtrates were stored at 4 °C. A second extraction was performed using 35 mL of the same extraction solvent in the dark for 72 h. After filtration with the Whatman filter paper, both filtrates were gathered and stored in normal conditions.

#### 3.4.2. Apparatus, Standard Curves, and Chromatographic Conditions

An external standard calibration method was used for quantitative determinations. Three manual injections of each standard (20 µL) at the concentrations listed in Table 6 were performed. The calibration curves were obtained by plotting the peak area (*y*) of the compound at each concentration level versus the sample concentration (*x*).

An Agilent 1200 High-Performance Liquid Chromatograph coupled to an Agilent UV-Vis diode array detector (Agilent Technologies, Santa Clara, CA, USA) was used for the chromatographic analysis. Six chromatographic methods were used to separate biomolecules on a Kinetex C18 column (4.6 × 150 mm, 5 µm, Phenomenex, Torrance, CA, USA), as listed in Table 7. Several mobile phases were used for biomarker identification, and UV spectra were recorded at different wavelengths, based on HPLC methods previously tested and validated for herbal medicines [29]. The identification of each biomarker (performed in triplicate) is based on their retention time and UV spectra compared to standard solutions.

### 3.5. In Vitro Antioxidant Activities

#### 3.5.1. DPPH Free Radical—Scavenging Capacity

The free radical removal capacity of methanol extracts was evaluated using the radical DPPH assay, described by Sreejayan and Rao [30], slightly modified. Briefly, 25 µL of leaf and stem methanol extracts of *U. bojeri* (7.8, 15.6, 31.25, 62.5, and 125 µg/mL) was added to 175 µL of methanol DPPH solution (0.25 mmol/L) in a 96-well microplate and incubated at room temperature for 30 min. Methanol was used as a blank and a methanol solution of DPPH was used as a negative control. Gallic acid (2.5, 5, 10, 20, and 40 µg/mL) was used as an antioxidant reference compound. Results were expressed as means of inhibiting concentration (IC), calculated using the following equation: IC (%) = 100 × (A_0_ – A_1_) / A_0_, where A_0_ and A_1_ are the values for the absorbance of the negative control and sample, respectively, at 517 nm. The IC_50_ (inhibition concentration at 50%) values of methanol extracts and gallic acid were calculated by linear regression (concentration of tested plant extracts or gallic acid vs. average percent of scavenging capacity by three replicates).

#### 3.5.2. Ferric Reducing Antioxidant Power (FRAP)

The process is based on the reduction of the ferric ions Fe^3+^ into ferrous ions Fe^2+^ in the 2,4,6-tripyridyl-s-triazine (TPTZ) complex [31]. The FRAP reagent was prepared, before analysis, by mixing TPTZ and FeCl_3_·6H_2_O solutions with acetate buffer (0.3 M). Then, 30 μL of the sample was mixed with 90 μL of distilled water and 900 μL of the FRAP reagent. The mixture was stirred and then incubated at 37 °C for 30 min. The absorbance was read at 595 nm using a UV-Vis spectrophotometer (UV–1600PC, VWR, Milan, Italy). The standard curve was obtained using FeSO_4_·7H_2_O with concentrations ranging from 100 to 1000 mmol/L, and the results were expressed as millimoles of ferrous ion equivalent per kilogram of dry weight.

### 3.6. Animals

Swiss albino male and female mice (weight: 25 ± 5 g; age: 4–5 months), kept under controlled conditions (12 h dark and 12 h light cycle, 25 ± 2 °C temperature, and 50% ± 10% humidity) at the *Institut Malgache de Recherches Appliquées* animal house, were used. The animals received a standard food pellet (1420, Livestock Feed Ltd., Port Louis, Mauritius) and they remained fasting for one night before the experiment. All experiments were carried out in accordance with the European Parliament and the Council of 22nd September 2010 on the protection of animals used for scientific purposes (DIRECTIVE 2010/63/EU). In this study, 140 mice were used. For this, research rats were not utilized, but mice were only tested. The number of consent of the Animal Ethics Committee was n° 04/CEA–IMRA/2019 (Supplementary Material).

### 3.7. Anti-Inflammatory Activity

#### 3.7.1. Carrageenan-Induced Paw Oedema

In vivo anti-inflammatory activity was evaluated based on the inhibition of a carrageenan-induced mouse hind paw oedema using a plethysmometer, as previously described by Buisseret et al. [32], slightly modified. Briefly, mice were fasted for 12 h with free access to water until the experiment. Fasted animals were divided into 10 groups of 5 mice. Group I orally received distillated water and served as the negative control. Group II, orally indomethacin-administered (10 mg/kg per os), was the positive control group, Groups III–VI were fed by leaf extracts, respectively, at doses of 50, 100, 200, and 400 mg/kg body weight (b.w.), and Groups VII–X were fed by stem extracts, respectively, at doses of 50, 100, 200, and 400 mg/kg b.w., one hour before the induction of 100 µl of carrageenan solution (2%) in normal saline solution (9%) into the right hind paw of each mouse. Paw volume was measured by a water plethysmometer (Ugo Basile 7140, Italy) before and 30, 60, 120, and 180 min after induction of carrageenan.

#### 3.7.2. Acetic Acid-Induced Writhing Test

The protocol of Olajide et al. [33] was slightly modified to determine the antinociceptive activity of the extracts. Ten groups of 5 fasted mice were used for performing the assays. Distilled water was orally administered to the negative control group (group I), while indomethacin at a dose of 10 mg/kg b.w. was administered, by gavage, to the positive control group (group II); stem methanol extracts at doses of 50, 100, 200, and 400 mg/kg b.w. were administered to groups III–VI, respectively; and leaf methanol extracts at doses of 50, 100, 200, and 400 mg/kg b.w. were administered to groups VII–X, respectively, one hour before the injection of 100 µL of acetic acid solution (1%) in 0.9% saline solution by the intraperitoneal route in order to induce characteristic writhing. The number of writhes occurring between 5 and 30 min after acetic acid injection was counted.

### 3.8. Anti-Diabetic Activity

Oral glucose tolerance tests were carried out as earlier described by Tombozara et al. [34] with slight modifications. Fasted mice were divided into ten groups (each group comprised four animals for each treatment): i) Group I, mice that received glibenclamide (GBD) at 10 mg/kg body weight (b.w.), 60 min before administration of 4 g/kg b.w. glucose (t = 0 min); ii) group II, mice that received distillated water (negative control), 60 min before administration of glucose (4 g/kg b.w.); iii) groups III–VI, mice that received, respectively, 50, 100, 200, and 400 mg/kg b.w. of leaf extracts, 60 min before administration of glucose (4 g/kg b.w.); iv) groups VII–X, mice that received, respectively, 50, 100, 200, and 400 mg/kg b.w. of stem extracts, 60 min before administration of glucose (4 g/kg b.w.). Methanol extracts or GBD were administrated by the oral route at the volume of 10 mL/kg. Glycaemia was measured from the venous blood sample taken at the end of the tail using a glucometer (Safe-Accu 2) at different periods (i) at t = −60 min, which is the time corresponding to the glycaemia before the extract or GBD administration; ii) at t = 0, which is the measured glycaemia, 60 min after the extract or GBD administration; iii) at t = 15, 30, 60, and 120 min, corresponding to the time after the glucose loading).

### 3.9. Statistical Analysis

All the experiments were carried out in triplicate and the results were expressed as mean ± standard deviation (SD) or standard error means (S.E.M.). Data were statistically analyzed using Student’s *t*-test and one-way analysis of variance (ANOVA) followed by Tukey’s HSD (honestly significant difference) multiple range test. All the differences showing a *p* < 0.05 were accepted as statistically significant.

## 4. Conclusions

This is the first phytochemical and pharmacological study on *U. bojeri*, and its importance is related to the wide use of this species in the popular Malagasy medicine. Indeed, despite its wide use by the Malagasy population, there is no information on the composition and the supposed benefits of this species for human health. Preliminary results highlighted, for the first time, that stem and leaf extracts of *U. bojeri* showed an anti-inflammatory, antidiabetic, and high antioxidant activity. This species is rich in secondary metabolites known for pharmacological activity. Thus, these first findings support the use of this species, in traditional medicine, but more studies are needed to better clarify pharmacological mechanisms of action.

## Figures and Tables

**Table 1:** Phytochemical fingerprint of analyzed samples.

Class	Standard	Stems (mg/100gDW)	Leaves (mg/100gDW)
Cinnamic acid	Caffeic acid	2.08 ± 0.07	0.77 ± 0.45
	Chlorogenic acid	n.d.	n.d.
	Coumaric acid	n.d.	n.d.
	Ferulic acid	n.d.	n.d.
Flavonols	Hyperosides	n.d.	77.94 ± 6.37
	Isoquercetrin	n.d.	n.d.
	Quercetin	n.d.	151.92 ± 13.35
	Quercitrin	n.d.	n.d.
	Rutin	n.d.	72.20 ± 7.97
Benzoic acids	Ellagic acid	335.14 ± 106.96	338.04 ± 123.79
	Gallic acid	n.d.	n.d.
Catechins	Catechin	n.d.	n.d.
	Epicatechin	n.d.	n.d.
Tannins	Castalagin	406.35 ± 223.20	943.83 ± 98.80
	Vescalagin	n.d.	n.d.
Monoterpenes	Limonene	n.d.	n.d.
	Phellandrene	n.d.	n.d.
	Sabinene	n.d.	0.21 ± 0.05
	ƴ-terpinene	n.d.	n.d.
	Terpinolene	n.d.	n.d.
Organic acids	Citric acid	n.d.	n.d.
	Malic acid	n.d.	n.d.
	Oxalic acid	n.d.	n.d.
	Quinic acid	n.d.	n.d.
	Succinic acid	533.74 ± 340.08	1275.65 ± 434.99
	Tartaric acid	n.d.	n.d.
Vitamins	Ascorbic acid	n.d.	n.d.
	Dehydroascorbic acid	n.d.	n.d.
Carotenoids	α-carotene	n.d.	n.d.
	β-carotene	n.d.	n.d.
	β-cryptoxanthine	0.78 ± 0.34	12.55 ± 6.54
	Lutein	2.85 ± 0.07	20.99 ± 1.34
	Lycopene	1.79 ± 0.95	1015.44 ± 253.01
	Zeaxanthine	n.d.	0.46 ± 0.03
TBCC		1281.72 ± 671.67	3909.01 ± 822.90

Each value represents the mean ± SD (standard deviation). DW = dry weight of the plant material; n.d. = not detected.

**Table 2:** Total phenolic content (TPC), ferric reducing antioxidant power (FRAP), and IC_50_ values of leaves and stems of *U. bojeri*.

Extract/Compound	TPC (mg of GAE/100 g of DW)	FRAP (mmol Fe^2+^/kg of DW)	DPPH Free Radical Scavenging Capacity		
			Linear Equation	R^2^	IC _50_ (μg/mL)
**Stems**	5854.17 ± 1247.65	70.17 ± 9.53	y = 1.2467x + 8.464	0.9819	33.32 ± 0.69 ^a,b^
**Leaves**	3624.72 ± 268.07	69.20 ± 1.41	y = 1.0731x − 0.8258	0.9901	47.36 ± 3.00 ^a^
**Gallic Acid**	-	-	y = 2.7182x − 6.3455	0.9928	20.73 ± 1.35

The data represent the mean ± SD of triplicate determinations of two experiments. SD = standard deviation; ^a^: *p* < 0.0001 vs. gallic acid; ^b^: *p* < 0.0001 vs. leaf extract.

**Table 3:** Effects of leaf and stem extracts of *U. bojeri* on carrageenan-induced mouse paw edema (n = 5).

Tested Substance	Dose (mg/kg)	Percent Anti-Inflammatory Activity (% A)			
		30 min	60 min	120 min	180 min
**Control**	-	0.77 ± 0.77	6.19 ± 1.76	21.28 ± 2.48	25.39 ± 6.68
**Indomethacin**	10	18.08 ± 0.77 ^a^	40.14 ± 8.08 ^b^	66.99 ± 7.74 ^a^	86.83 ± 5.54 ^a^
**Leaf Extract**	50	6.94 ± 1.40	15.46 ± 1.47 ^b^	32.44 ± 2.10 ^b^	44.66 ± 2.69 ^b^
	100	6.04 ± 1.28	17.33 ± 1.33 ^b^	29.36 ± 2.27 ^c^	46.89 ± 2.80 ^b^
	200	23.94 ± 2.01 ^c^	43.80 ± 4.31 ^a^	62.09 ± 3.33 ^a^	77.43 ± 2.49 ^a^
	400	28.77 ± 1.64 ^b^	44.71 ± 2.71 ^a^	61.16 ± 1.94 ^a^	83.62 ± 4.41 ^a^
**Stem Extract**	50	10.35 ± 0.38 ^b^	19.27 ± 1.34 ^a^	29.01 ± 1.92 ^c^	41.42 ± 0.80 ^b^
	100	11.06 ± 2.44 ^c^	23.61 ± 1.32 ^a^	38.78 ± 1.65 ^b^	53.67 ± 1.90 ^a^
	200	16.35 ± 1.31 ^a^	29.23 ± 2.28 ^a^	53.19 ± 1.84 ^a^	65.47 ± 1.69 ^a^
	400	18.70 ± 3.50 ^a^	32.04 ± 1.43 ^a^	53.39 ± 1.37 ^a^	71.59 ± 3.21 ^a^

The data represent the mean ± S.E.M. (standard error means). ^a^: *p* < 0.001; ^b^: *p* < 0.01; ^c^: *p* < 0.05 vs. control. stems *p* < 0.001 t 60, 120, 180 ANOVA followed by Tukey’ Post Hoc. leaves *p* < 0.001 t 30, 60, 120, 180 ANOVA followed by Tukey Post Hoc.

**Table 4:** Effect of leaf and stem extracts of *U. bojeri* on nociceptive responses in the acetic acid-induced writhing test.

Treatment	Dose (mg/kg)	Number of Writhes (5–25 min)	Inhibition (%)
**Control**	-	24.90 ± 2.25	0
**Indomethacin**	10	3.90 ± 1.12 ^a^	84.34
**Leaf Extract**	50	19.00 ± 1.15 ^c^	23.70
	100	16.70 ± 0.82 ^b^	32.93
	200	13.20 ± 0.72 ^b^	46.99
	400	12.60 ± 1.34 ^b^	49.40
**Stem Extract**	50	19.70 ± 1.64	20.88
	100	15.50 ± 0.67 ^b^	37.75
	200	12.30 ± 1.70 ^b^	50.60
	400	10.60 ± 1.22 ^b^	57.43

The data represent the mean ± S.E.M. (standard error means). ^a^: *p* < 0.001; ^b^: *p* < 0.01; ^c^: *p* < 0.05 vs. control. ANOVA *p* < 0.01 leaves & stems.

**Table 5:** Effect of methanol extracts of *U. bojeri* leaves and stems on mice blood glucose level (mmoles/L).

Group (n = 4)	Dose (mg/kg)	Time (min)				
		0	15	30	60	120
**Negative Control**	-	6.21 ± 0.11 0	23.13 ± 0.83 16.92	18.67 ± 0.42 12.46	12.25 ± 0.86 6.04	6.95 ± 0.29 0.74
**Leaf Extract**	50	5.62 ± 0.29 0	24.54 ± 1.18 18.92	17.14 ± 0.69 11.52	11.87 ± 0.54 6.25	6.28 ± 0.58 0.66
	100	6.22 ± 0.18 0	24.02 ± 0.78 17.80	16.10 ± 0.68 ^c^ 9.99	11.77 ± 0.71 5.55	6.40 ± 0.28 0.18
	200	5.72 ± 0.21 0	26.04 ± 1.07 20.32	12.11 ± 0.70 ^a^ 6.39	8.87 ± 0.60 ^c^ 3.15	5.78 ± 0.16 ^c^ 0.06
	400	6.12 ± 0.04 0	25.04 ± 0.36 18.92	10.89 ± 0.89 ^a^ 4.77	7.62 ± 0.31 ^b^ 1.5	5.63 ± 0.15 ^b^ –0.49
**Stem Extract**	50	6.00 ± 0.19 0	23.29 ± 1.01 17.29	17.14 ± 0.70 11.14	12.31 ± 0.48 6.31	6.76 ± 0.38 0.76
	100	6.22 ± 0.17 0	24.50 ± 1.06 18.28	15.55 ± 0.42 ^b^ 9.33	12.02 ± 0.82 5.80	6.25 ± 0.21 0.03
	200	5.82 ± 0.41 0	24.54 ± 1.00 18.72	11.66 ± 0.51 ^a^ 5.84	9.36 ± 0.16 ^c^ 3.54	5.90 ± 0.27 ^c^ 0.08
	400	6.12 ± 0.04 0	25.04 ± 1.11 18.92	10.39 ± 0.30 ^a^ 4.27	8.35 ± 0.22 ^b^ 2.23	5.74 ± 0.09 ^b^ –0.38
**Glibenclamide**	10	5.25 ± 0.37 0	25.22 ± 2.18 19.97	12.71 ± 0.90 ^b^ 7.46	7.42 ± 0.39 ^b^ 2.17	6.58 ± 0.30 1.33

Each top value is the mean ± S.E.M. (standard error means) of blood glucose level of four separated mice. Each bottom value represents the augmentation of blood glucose level related to those of 0 min. ^a^: *p* < 0.001 vs. negative control; ^b^: *p* < 0.010 vs. negative control; ^c^: *p* < 0.050 vs. negative control. Leaves & stems *p* < 0.05 with ANOVA at t 30 and 60 followed by Tukey Post Hoc.

**Table 6:** Calibration curve, R^2^, LOD (limit of detection), and LOQ (limit of quantification) of each bioactive compound used as standard.

Chromatographic Method	Class	Standard	Retention Time (min)	Wavelength (nm)	Calibration Curve Equation	R^2^	Calibration Curve Range (mg·L^−1^)	LOD (mg·L^−1^)	LOQ (mg·L^−1^)
**A**	Cinnamic acid	Caffeic acid	4.54	330	y = 59.046x + 200.6	0.996	111–500	0.305	1.016
		Chlorogenic acid	3.89	330	y = 15.583x + 760.05	0.984	111–500	0.940	3.134
		Coumaric acid	6.74	330	y = 8.9342x + 217.4	0.997	111–500	2.907	9.690
		Ferulic acid	7.99	330	y = 3.3963x − 4.9524	1.000	111–500	1.245	4.150
	Flavonols	Hyperosides	10.89	330	y = 7.1322x − 4.583	0.999	111–500	3.372	11.241
		Isoquercetrin	11.24	330	y = 8.3078x + 26.621	0.999	111–500	0.252	0.840
		Quercetin	17.67	330	y = 3.4095x − 98.307	0.998	111–500	4.055	13.518
		Quercitrin	13.28	330	y = 2.7413x + 5.6367	0.998	111–500	5.456	18.187
		Rutin	12.95	330	y = 6.5808x + 30.831	0.999	111–500	2.937	9.790
**B**	Benzoic acids	Ellagic acid	18.65	280	y = 29.954x + 184.52	0.998	62.5–250	0.611	2.035
		Gallic acid	4.26	280	y = 44.996x + 261.86	0.999	62.5–250	0.435	1.451
	Catechins	Catechin	10.31	280	y = 8.9197x + 66.952	1.000	62.5–250	2.343	7.809
		Epicatechin	14.30	280	y = 12.88x − 43.816	0.999	62.5–250	0.763	2.543
	Tannins	Castalagin	16.35	280	y = 4.236x − 8.535	1.000	62.5–250	1.009	3.363
		Vescalagin	17.25	280	y = 4.939x − 1.232	1.000	62.5–250	0.603	2.010
**C**	Monoterpenes	Limonene	3.35	250	y = 0.189x − 5.420	0.999	125–1000	8.654	28.847
		Phellandrene	3.57	210	y = 8.783x − 145.3	0.998	125–1000	0.562	1.874
		Sabinene	3.45	220	y = 18.14x − 1004	0.998	125–1000	0.094	0.314
		ƴ-terpinene	3.28	235	y = 0.4886x − 23.02	0.999	125–1000	15.577	58.590
		Terpinolene	4.83	220	y = 26.52x + 876.8	0.999	125–1000	0.241	0.804
**D**	Organic acids	Citric acid	5.30	214	y = 1.0603x − 22.092	1.000	167–1000	18.805	62.682
		Malic acid	4.03	214	y = 1.415x − 80.254	0.996	167–1000	15.721	52.404
		Oxalic acid	7.85	214	y = 6.4502x + 6.1503	0.998	167–1000	0.550	1.835
		Quinic acid	3.21	214	y = 0.8087x − 38.021	0.998	167–1000	26.106	87.021
		Succinic acid	3.46	214	y = 0.9236x + 8.0823	0.995	167–1000	7.135	23.783
		Tartaric acid	5.69	214	y = 1.8427x − 15.796	1.000	167–1000	8.520	28.401
**E**	Vitamins	Ascorbic acid	4.14	261	y = 42.71x + 27.969	0.999	100–1000	0.836	2.786
		Dehydroascorbic acid	3.41	348	y = 4.1628x + 140.01	0.999	30–300	1.095	3.649
**F**	Carotenoids	α-carotene	12.34	450	y = 0.5323x + 4.2783	0.994	25–100	1.546	5.154
		β-carotene	10.58	450	y = 1.5762x + 1.8981	0.992	25–250	3.976	13.254
		β-cryptoxanthin	4.35	450	y = 13.272x − 5.3181	0.999	25–200	0.305	1.016
		Lutein	2.33	450	y = 84.448x − 318.78	1.000	25–200	0.073	0.244
		Lycopene	3.16	450	y = 0.8543x + 19.263	0.979	62.5–500	14.933	49.775
		Zeaxanthin	2.43	450	y = 188.23x − 56.64	1.000	25–200	0.042	0.141

**Table 7:** Chromatographic conditions of each used method.

Method	Compound of Interest	Stationary Phase	Mobile Phase	Flow (mL/min)	Time of Analysis (mn)	Gradient ^b^	Wavelength (nm)
**A**	Cinnamic acids, flavonols	KINETEX-C18 column (4.6 × 150 mm, 5 μm)	A: 10 mM KH_2_PO_4_/H_3_PO_4_, pH = 2.8 B: CH_3_CN	1.5	20 + 2(CT) ^a^	Yes	330
**B**	Benzoic acids, catechins	KINETEX-C18 column (4.6 × 150 mm, 5 μm)	A:H_2_O/CH_3_OH/HCOOH (5:95:0.1 v/v/v), pH = 2.5 B: CH_3_OH/HCOOH (100:0.1 v/v)	0.6	23 + 2(CT) ^a^	Yes	280
**C**	Monoterpenes	KINETEX-C18 column (4.6 × 150 mm, 5 μm)	A: H_2_O B: CH_3_CN	1.0	17 + 3(CT) ^a^	Yes	210,220,235,250
**D**	Organic acids	KINETEX-C18 column (4.6 × 150 mm, 5 μm)	A: 10 mM KH_2_PO_4_/H_3_PO_4_, pH = 2.8 B: CH_3_CN	0.6	13 + 2(CT) ^a^	No	214
**E**	Vitamins	KINETEX-C18 column (4.6 × 150 mm, 5 μm)	A: 5 mM C_16_H_33_N(CH_3_)3Br/50 mM KH_2_PO_4_, pH = 2.5 B: CH_3_OH	0.9	10 + 5(CT) ^a^	No	261,348
**F**	Carotenoids	KINETEX-C18 column (4.6 × 150 mm, 5 μm)	A: ACN B: MeOH C: CH_2_Cl_2_	1.0	20 + 5(CT) ^a^	No	450

^a^ CT = conditioning time. ^b^ Elution conditions. Method A gradient: 5% B to 21% B in 17 min + 21% B in 3 min. Method B gradient: 3% B to 85% B in 22 min + 85% B in 1 min. Method C ratio of phase A and B: 95:5. Method D ratio of phase A and B: 95:5. Method E ratio of phase A and B: 95:5. Method F ratio of phase A, B, and C: 75:20:5.

## Supplementary Materials

The following are available online at https://www.mdpi.com/1424-8247/13/4/71/s1, Supplementary material: Consent of the Animal Ethics Committee—n° 04/CEA–IMRA/2019.
