# Supplementary material for: Antioxidant, Anti-Inflammatory, and Antidiabetic Activities of Leaves and Stems of Uapaca bojeri Bail. (EUPHORBIACEAE), an Endemic Plant of Madagascar
Source: Pharmaceuticals (Basel). 2020 Apr 17;13(4):71. doi: 10.3390/ph13040071 (PMC7243096; doi:10.3390/ph13040071)
Supplement: Supplementary file 1 [file pharmaceuticals-13-00071-s001.pdf]

INSTITUT MALGACHE DE RECHERCHES APPLIQUEES
Fondation Suzanne et Albert RAKOTO RATSIMAMANGA
Fondation reconnue d'utilité publique
Membre de l'Agence Universitaire de la Francophonie
Médecine traditionnelle et Nutrition

Objet : Décision du comité d'éthique animale de l'IMRA (n° 04/CEA-IMRA/2019) relative à la demande formulée par Monsieur RAZAFINDRAKOTO Zoazilala Rinah concernant ses protocoles d'étude intitulée « Etude des activités anti-inflammatoire, antalgique et hypoglycémiante des extraits bruts méthanoliques des feuilles et d'écorce de tige de *Uapaca bojeri* (EUPHORBIACEES) chez la souris ».

Le comité d'éthique animale de l'Institut Malgache de Recherches Appliquées, après avoir étudié les protocoles susmentionnés reçus le vendredi 05 avril 2019 et après avoir écouté et discuté avec le demandeur le vendredi 12 avril 2019, a constaté que les protocoles proposés respectent les recommandations du Parlement et du Consul Européens du 22 septembre 2010 sur la protection des animaux utilisés à des fins scientifiques (Directive 2010/63/EU). En conséquence, le comité émet « **un avis favorable** » à la réalisation de cette étude.

Les membres ont suggéré à Monsieur Razafindrakoto Zoarilala de réduire le nombre d'animaux par lot à 5 pour respecter les règles de 3R (replace, reduce and refine).

FONDATION Albert et Suzanne RAKOTO RATSIMAMANGA
Avarabohitra
IMRA
Antananarivo

Fait à Antananarivo, ce 19 avril 2019.

Pr RAMANITRAHASIMBOLA David, pharmacologue
Président du Comité d'Ethique Animale, IMRA

INSRE : 972/02/8053963 - BP 3833 - Antananarivo 101, Madagascar
Tel : 261 20 22 381 88 ; Fax : 261- 20 22 304 70
e-mail : soamadi@wanadoo.mg
